# Patients with hypertrophic cardiomyopathy (HCM) and HCM gene carriers have attenuated myocardial oxygenation response to vasodilator stress - a potential mechanism for sudden cardiac death

**DOI:** 10.1186/1532-429X-14-S1-P157

**Published:** 2012-02-01

**Authors:** Sairia Dass, Theodoros Karamitsos, Joseph Suttie, Emily Sever, Michael Jerosch-Herold, Hugh Watkins, Stefan Neubauer

**Affiliations:** 1 Deprtment of Cardiovascular Medicine, University of Oxford, Oxford, UK; 2 Radiology, Brigham and Women's Hospital, Boston, MA, USA

## Summary

Tissue oxygenation response to adenosine stress in patients with hypertrophic cardiomyopathy (HCM) is blunted compared to athletes and normals. Myocardial tissue hypoxia during stress in HCM may potentially contribute to the increased exercise related deaths in this condition.

## Background

By exploiting the paramagnetic properties of deoxyhemoglobin, blood oxygen level-dependent (BOLD) MRI can detect myocardial ischemia in patients with coronary artery disease. However, little is known about myocardial tissue oxygenation in pathological left ventricular hypertrophy (e.g. HCM) or physiological hypertrophy (e.g. elite athletes). Perfusion studies have shown that patients with HCM show evidence of microvascular dysfunction, however, whether this leads to de-oxygenation and ischemia is unclear.

## Methods

Sixty nine age and gender matched subjects (26 HCM; 11 HCM gene carriers without hypertrophy; 12 athletes; 20 normal controls) were studied at 3 Tesla (Siemens Tim Trio), with acquisition of BOLD (using aT2-prepared sequence) and first-pass perfusion images (using a saturation recovery fast-gradient echo sequence and 0.03 mmol/kg Gd-DTPA bolus) at stress and rest (4-6 minutes i.v. adenosine, 140μg/kg/min). Signal intensity change (SIΔ) and myocardial perfusion reserve index (MPRI) were measured from BOLD and perfusion images, respectively.

## Results

During stress there were equivalent rises in rate pressure product in all groups, (normal 73±20%, HCM 74±50%, athlete 80±38%, P=NS).

**Figure 1:**
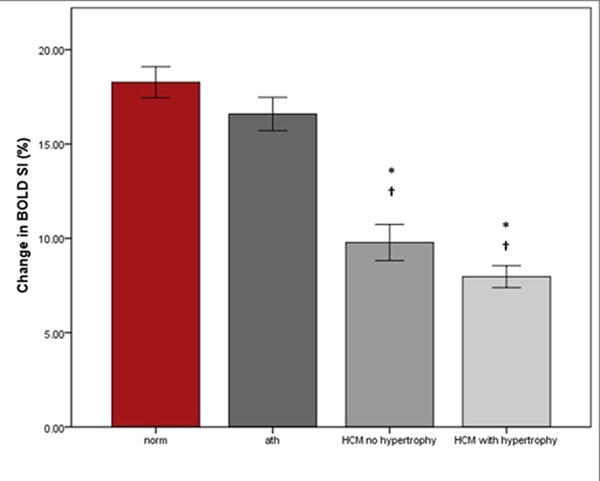
The mean and standard deviation of BOLD SIΔ% with adenosine stress in normal, athletes, and HCM patients with and without hypertrophy. Error pars: standard error. * P<0.001 compared to normal; †P<0.001 compared to athletes.

**Figure 2:**
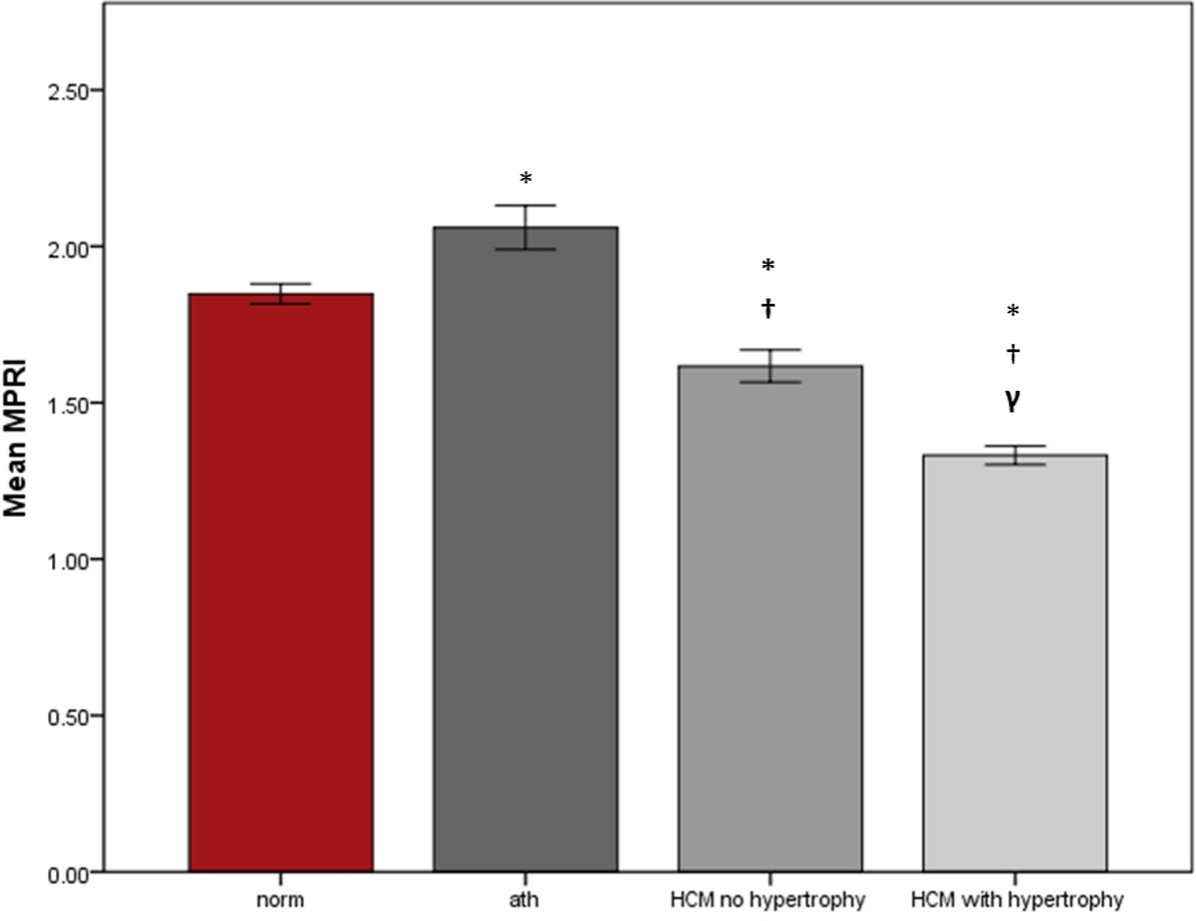
The mean and standard deviation of MPRI with adenosine stress in normal, athlete, HCM with and without hypertrophy. Error pars: standard error. * P<0.001 compared to normal; †P<0.001 compared to athletes; γ P<0.05 compared to HCM with no hypertrophy.

There was a significantly reduced BOLD SIΔ response in both HCM gene carriers and HCM (BOLD SIΔ: 10±11% gene carriers; 8±10% HCM P<0.0001 vs normal, figure 1) compared to athletes (17±10%) and normal volunteers (18±14% P=0.8). MPRI was also significantly reduced in HCM, (normal controls: 1.8±0.6; athletes: 2.0±0.9, P=0.005; HCM gene carriers: 1.6±0.7 P=0.001; HCM: 1.3 ± 0.6 P<0.0001, figure 2). There was a weak but significant correlation between BOLD SIΔ and MPRI per segment, (R=0.27, P<0.0001) and between BOLD SIΔ and end diastolic wall thickness per segment, (R= 0.24, P< 0.001). Linear regression analysis showed that both MPRI (β 0.2, P<0.001) and wall thickness (β-0.2, P<0.001) are independent predictors of BOLD SIΔ.

## Conclusions

Patients with HCM as well as HCM gene carriers without evidence of hypertrophy show blunted myocardial oxygenation response to vasodilator stress compared to normal controls and athletes. The increased energy cost, and thus oxygen demand, of contraction in HCM, regardless of the degree of phenotype expression, together with barriers to oxygen supply due to the impaired perfusion are the most likely pathophysiological mechanisms of de-oxygenation in these patients. Importantly, myocardial tissue hypoxia may play a significant pathophysiological role being potentially responsible for stress-induced arrhythmia and sudden death in HCM.

## Funding

This research is funded by the British Heart Foundation.

